# Coupling of Nanoporous Chromium, Aluminium-Containing Silicates with an Ionic Liquid for the Transformation of Glucose into 5-(Hydroxymethyl)-2-furaldehyde

**DOI:** 10.3390/molecules17043690

**Published:** 2012-03-26

**Authors:** Margarida M. Antunes, Sérgio Lima, Martyn Pillinger, Anabela A. Valente

**Affiliations:** Department of Chemistry, CICECO, University of Aveiro, 3810-193 Aveiro, Portugal; Email: margarida.antunes@ua.pt (M.M.A.); sergiolima@ua.pt (S.L.); mpillinger@ua.pt (M.P.)

**Keywords:** glucose, 5-(hydroxymethyl)-2-furaldehyde, ionic liquid, solid acids

## Abstract

Micro/mesoporous chromium, aluminium-containing silicates of the type TUD-1 (Al-TUD-1, Cr-TUD-1, CrAl-TUD-1) and zeolite BEA, Cr-BEA, and related composites BEA/TUD-1 and Cr-BEA/TUD-1, were prepared, characterised, and tested as solid acids coupled with the ionic liquid (IL) 1-butyl-3-methylimidazolium chloride ([bmim]Cl) as solvent, in the transformation of d-glucose into 5-(hydroxymethyl)-2-furaldehyde (Hmf), at 120 °C. The chromium-containing catalytic systems lead to considerably higher Hmf yields in comparison to the related systems without chromium. The IL is a favourable solvent for this target reaction (in terms of Hmf yields reached) compared to water or dimethylsulfoxide. A detailed study on the stabilities of the nanoporous solid acids in the IL medium is presented.

## 1. Introduction

Lignocellulosic matter is a promising renewable source for the sustainable production of chemicals, materials and fuels which are presently produced essentially from fossil fuels, and is obtainable from agricultural and forestry residues/surpluses, waste streams of biorefineries and municipal paper waste [1,2]. Lignocellulosic biomass is mainly composed of the carbohydrate polymers, cellulose and hemicelluloses. Cellulose is the most abundant terrestrial biopolymer and consists of chains of D-anhydroglucopyranose residues linked by β-1,4-glycosidic bonds which are hydrolysable under acidic reaction conditions to give the monosaccharide, d-glucose (Glu, a hexose). Glu is thus the major building block obtained from carbohydrate biomass, and can be converted into promising platform chemicals such as 5-(hydroxymethyl)-2-furaldehyde (Hmf) with a wide applications profile [3].

The reaction of Glu to Hmf is rather demanding in comparison to, for example, fructose (hexose), partly due to the fact that the reaction mechanism is complex, involving several elementary steps with different acid-base requirements. Zhao *et al.* reported one of the most effective catalytic systems known to date for the selective conversion of Glu to Hmf, consisting of chromium salts as homogeneous Lewis acid catalysts dissolved in an IL solvent, under relatively mild reaction conditions [4,5,6]; CrCl_2_ coupled with 1-ethyl-3-methyl imidazolium chloride ([emim]Cl) led to ca. 70% Hmf yield at 95% conversion (10 wt.% glucose, 100 °C, 3 h reaction) [4]. Since then, several chromium/IL based catalytic systems have been successfully investigated in the conversion of Glu (and related di/polysaccharides) to Hmf, generally exhibiting superior catalytic performances in comparison to IL-based catalytic systems containing other transition metals [7,8,9]. The use of an IL as solvent is favourable for this target reaction. Relatively low Hmf yields have been reported for chromium salts (CrCl_2_, CrSO_4_) used as catalysts in the aqueous phase reaction of Glu, at 140 °C [10], or of cellulose at 180 °C [11]. The use of ILs as solvents instead of water avoids Hmf-loss reactions, typically occurring in aqueous media [12]. On the other hand, some ILs are favourable (“non-volatile”) solvents for dissolving carbohydrates such as crystalline cellulose, in comparison to water and most common organic solvents (important for process intensification) [13,14,15,16,17,18,19], and these types of solvents can be obtained from renewable resources [20].

Homogeneous catalytic systems require careful work-up procedures in order to avoid losses of catalyst and contamination of effluents (requiring demanding/costly separation/purification processes and treatment/disposal of waste streams). The coupling of solid acid catalysts with ILs as solvents is a possible approach to minimize these drawbacks. Only a couple of studies can be found in the literature reporting on the reaction of Glu (or related di/polysaccharides) to Hmf using (chromium-containing solid acid)/IL catalytic systems. Physical mixtures of chromium salts and solid acids (zeolites H-Y, H-BEA, H-MOR, H-ZSM-5, or acidic Amberlyst ion-exchange resins) coupled with [bmim]Cl as IL solvent have been investigated in the conversion of cellulose to Hmf, at 120 °C, and the best result was ca. 36% Hmf yield at 6 h reaction using H-Y zeolite as solid acid [21]. In a recent study it was reported that the addition of ILs to aqueous solutions of Glu enhanced adsorption of the latter on zeolites [22]. Interesting results were reported for hydroxyapatite-supported chromium chloride (Cr-HAP) coupled with [bmim]Cl as solvent in that this catalytic system was reused four times without significant decrease in the Hmf yield (ca. 40% yield in 2.5 min, using the microwave heating method, 400 W) [23]. A critical issue is the stability of the solid acids in the IL medium. According to the literature, Brönsted solid acids may undergo ion-exchange reactions in the IL medium, and the catalytic reactions may be effectively homogeneous in nature [24,25,26]. In the case of the Cr-HAP/IL system the homo/ heterogeneous nature of the catalytic reaction was not assessed [23].

The three-dimensional sponge-like mesoporous material TUD-1 is straightforward to prepare via (relatively low-cost) non-surfactant templating routes [24,27]. The high specific surface area, pore widths and volumes, and the 3-D channel system are favorable factors for the internal diffusion of relatively bulky reactant molecules and accessibility to the active sites. The purely siliceous TUD-1 may be furnished with Brönsted and Lewis acidity by the incorporation of metals such as Al and Cr into the framework via a one-pot procedure based on the sol-gel technique [28,29,30,31]. We have reported that Al-TUD-1 is an active and stable catalyst for the aqueous-phase dehydration of saccharides to furanic aldehydes, although the hexose-based mono/disaccharides gave less than 20% Hmf yield [32].

In this work, nanoporous chromium, aluminium-containing silicates were tested as solid acids in the reaction of Glu, using the ionic liquid [bmim]Cl as solvent, at 120 °C. The investigated solid acids are: mesoporous TUD-1 type materials possessing chromium and/or aluminium (Al-TUD-1, CrAl-TUD-1, Cr-TUD-1); zeolite BEA in the H^+^ form, Cr-BEA (prepared from BEA via ion-exchange reaction), and related micro/mesoporous composite materials BEA/TUD-1 and Cr-BEA/TUD-1. The stabilities of the nanoporous materials in the IL medium and the influence of the type of solvent ([bmim]Cl *versus* water or dimethylsulfoxide) on the reaction of Glu were investigated.

## 2. Results and Discussion

### 2.1. Synthesis and Characterisation of the Catalysts

Table 1 shows the chemical composition of the prepared materials. For Al-TUD-1 and CrAl-TUD-1 the atomic ratios agree roughly with those used in the respective synthesis mixtures; for Cr-TUD-1 the Si/Cr ratio of 150 is higher than the value of 100 used in the synthesis, suggesting that a fraction of the initial amount of chromium was not incorporated in the mesoporous silicate. 

**Table 1:** Physicochemical properties of the prepared materials ^a^.

Sample	Si/Al	Si/Cr	S_BET_ (m^2^·g^−1^)	V_p_ (cm^3^·g^−1^)	PSD (pore width, nm)
Al-TUD-1	20	-	726 (789)	0.64 (0.69)	2.5–7 (2.5–7)
CrAl-TUD-1	27	108	763 (995)	0.78 (0.95)	2.5–7 (2.5–7)
Cr-TUD-1	-	150	484 (515)	1.02 (1.14)	3–17 (3–17)
BEA	12	-	634	0.74	-
Cr-BEA	13	47	702 (793)	0.87 (0.75)	-
BEA/TUD-1	31	-	685 (722)	0.78 (0.82)	2.5–10 (2.5–10)
Cr-BEA/TUD-1	41	236	802 (717)	0.92 (0.80)	2.5–10 (2.5–10)

^a^ The values in brackets refer to the recovered solids.

**Figure 1:**
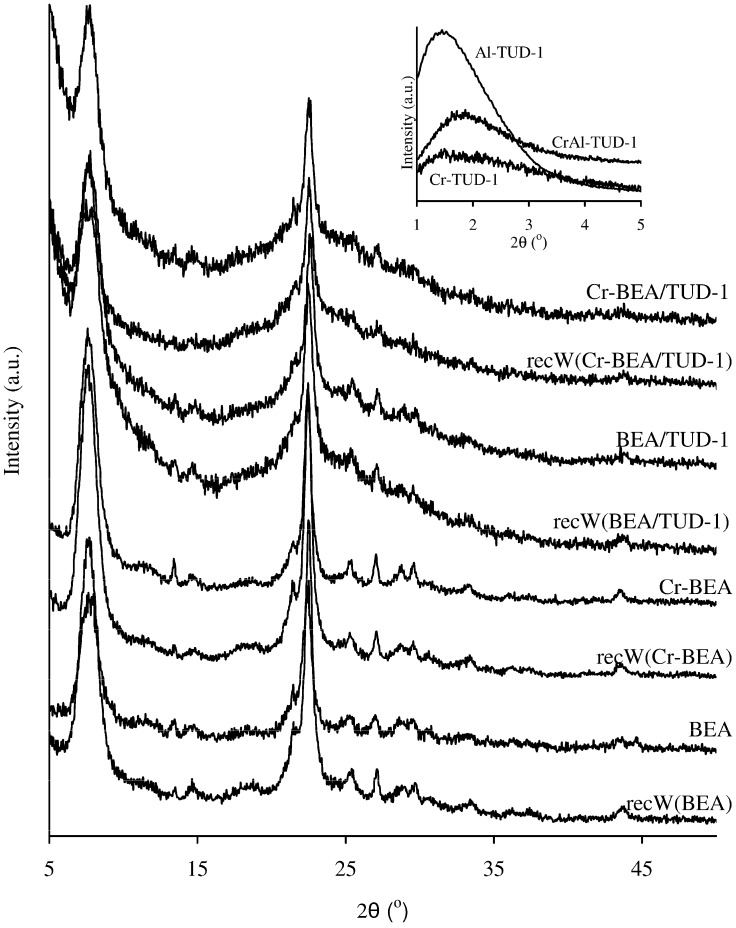
Powder XRD patterns of the fresh zeolite BEA-related materials and of the respective recW solids. The inset shows the low angle powder XRD patterns of the remaining materials prepared.

**Figure 2:**
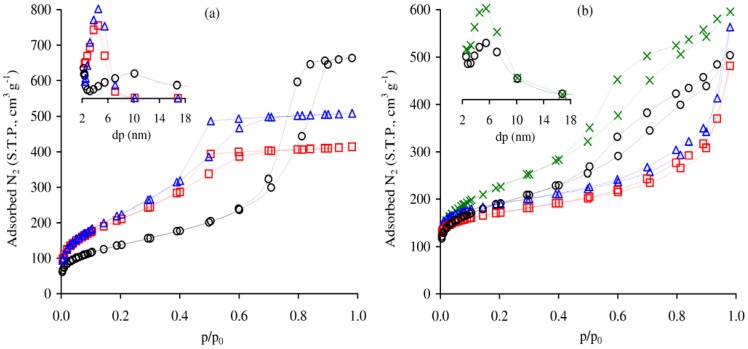
N_2_ adsorption-desorption isotherms at −196 °C of (**a**) Al-TUD-1 (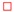), CrAl-TUD-1 (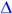), Cr-TUD-1 (○), and (**b**) BEA (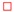), Cr-BEA (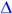), BEA/TUD-1 (○), Cr-BEA/TUD-1 (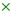). The insets show the PSD curves (dp is the pore width) for the respective materials (with matching symbols).

The powder X-ray diffraction (XRD) patterns for Al-TUD-1, Cr-TUD-1 and CrAl-TUD-1 showed one broad peak at low angles (ca. 1.5° 2*θ* for Cr-TUD-1 and CrAl-TUD-1, and 1.8° 2*θ* for Al-TUD-1 (inset of Figure 1) and a very broad peak centred around 25° 2*θ* (not shown), indicating that these materials are amorphous, but have mesostructured material characteristics [28,29,30,32,33,34]. No evidence of crystalline phases (e.g., alumina or chromium oxides) was detected in the patterns. These materials exhibit type IV N_2_ adsorption-desorption isotherms at −196 °C, with a H2 hysteresis loop (Figure 2a), which is consistent with an interconnected (worm-like) mesoporous network characteristic of TUD-1 type materials [30,31,32,35,36,37]. The capillary condensation in the mesopores occurs in the relative pressure range of about 0.4–0.6 for Al-TUD-1 and CrAl-TUD-1, and 0.5–0.8 for Cr-TUD-1, above which the adsorption branch levels off (suggesting that the external surface area is minor). Similar results were reported previously for metal-incorporated TUD-1 samples [28,29,30,31]. The texture properties of Al-TUD-1 and CrAl-TUD-1 are similar; in comparison, the S_BET_ for Cr-TUD-1 is lower, and the PSD is wider and for greater values of pore widths (Table 1, inset of Figure 2a).

The zeolite BEA and the related ion-exchanged Cr-BEA possess similar Si/Al ratios; in the latter case, the Si/Cr ratio is 47. Based on the aluminium contents it is possible to estimate that the BEA/TUD-1 and Cr-BEA/TUD-1 composites possess ca. 26 wt.% BEA and 33 wt.% Cr-BEA, respectively. In the case of the chromium-containing materials, the Al/Cr ratio is slightly higher for Cr-BEA/TUD-1 than for Cr-BEA (6 and 4, respectively), possibly due to partial leaching of chromium from BEA during the preparation of Cr-BEA/TUD-1. The XRD diffraction patterns of BEA and Cr-BEA are similar suggesting that the crystalline structure was preserved during the ion-exchange procedure (Figure 1). The XRD patterns of BEA and the related composite BEA/TUD-1 are similar, showing the characteristic diffraction peaks of zeolite Beta at 2*θ* = 7–8° and 22.5° [38]; the same applies when comparing the data for Cr-BEA and Cr-BEA/TUD-1.

The sorption isotherms for BEA and Cr-BEA show a significant increase in N_2_ uptake at *p*/*p*_0_ < 0.01, typically associated with the filling of micropores, followed by a gradual increase in N_2_ uptake as relative pressures approach unity (Figure 2), most likely due to multilayer adsorption on the external surface of the nanocrystallites. The related composite materials BEA/TUD-1 and Cr-BEA/TUD-1 exhibit type IV isotherms (Figure 2), typical of mesoporous materials, with a hysteresis loop at *p*/*p*_0_ > 0.4, which is associated with capillary condensation/evaporation in mesopores; for the two materials the PSD curves lie in the range of pore widths 2.5–10 nm (Table 1).

**Figure 3:**
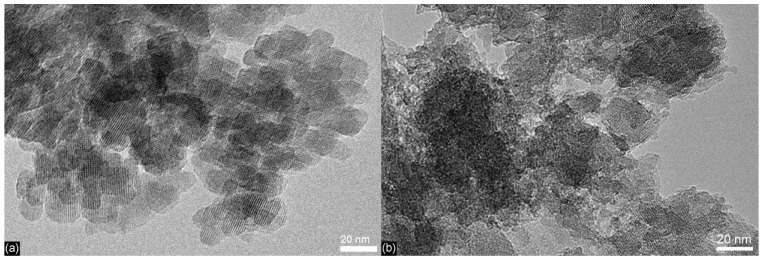
High-resolution TEM images of (**a**) Cr-BEA and (**b**) Cr-BEA/TUD-1.

The high-resolution (HR) TEM images of Cr-BEA (exemplified in Figure 3a) showed small crystallites with a size of about 20–30 nm and the lattice fringes characteristic of zeolite Beta. In the case of Cr-BEA/TUD-1, HRTEM characterisation showed a three-dimensional sponge- or worm-like mesoporous matrix with some dark gray domains that may be attributed to the embedded zeolite particles (Figure 3b), suggesting that Cr-BEA/TUD-1 is a composite of an amorphous mesoporous matrix and nanocrystalline Beta particles (isolated nanocrystals of ca. 20–30 nm, or aggregates of ca. 50–200 nm), which are fairly evenly distributed nanocrystals in the surrounding mesoporous matrix. The presence of small aggregates is in agreement with previous findings for a Beta/TUD-1 composite material with a zeolite loading of 40 wt.% [38,39], and can be attributed to the combined effect of the synthesis conditions and the high zeolite loading. These results are comparable to those previously reported by us for a composite material consisting of zeolite Beta and TUD-1 prepared in a similar fashion (and using the same commercial zeolite ammonia Beta powder) to that used in the present work [39].

**Figure 4:**
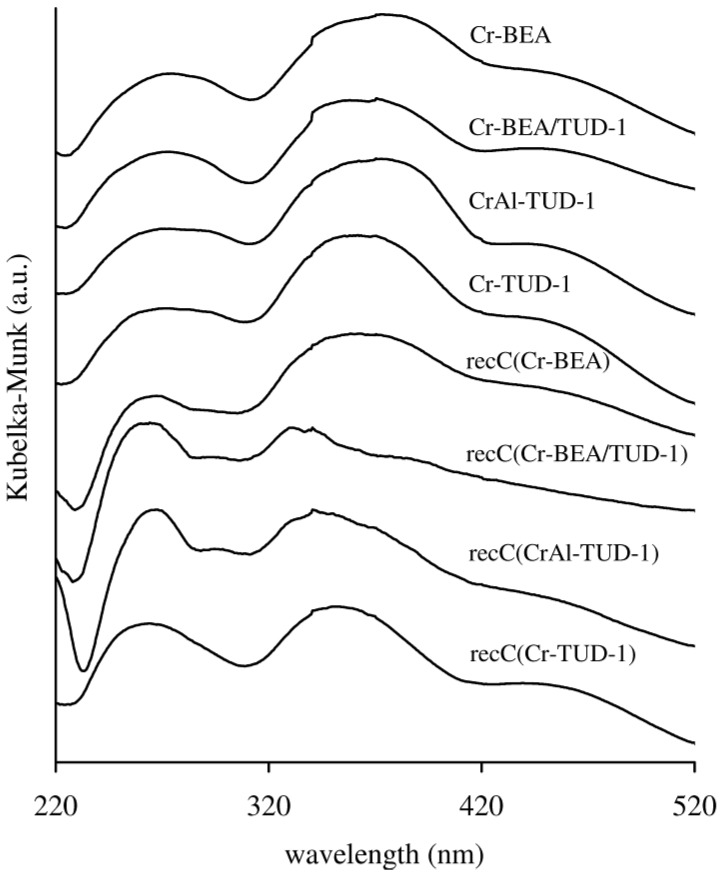
Diffuse reflectance UV-vis spectra of the chromium-containing materials and the related recC solids.

The yellow colour of all the chromium-containing samples suggests that the major species present was monochromate [40]. Accordingly, two very broad bands at ca. 275 (220–320 nm) and 365 (320–420 nm) in the DR UV-vis spectra (Figure 4) are assigned to O^2−^→Cr^6+^ charge transfer transitions of tetrahedrally coordinated isolated Cr^VI^ [30,40,41,42,43]. Similar results were reported previously for Cr-TUD-1 [30] and chromium-substituted BEA [42,43]. The shoulder at 450 nm (420–520 nm) may be due to dichromate or polychromate species [40,41], or, as proposed previously for Cr-TUD-1 [30], distorted isolated chromate species. The assignment of this band to octahedral Cr^III^ species (including Cr_2_O_3_-like clusters) can be excluded since no bands were detected at wavelengths greater than 600 nm, where octahedral Cr^III^ would be expected [30,41,42,43].

### 2.2. Catalysis

#### 2.2.1. General Considerations

**Scheme 1:**
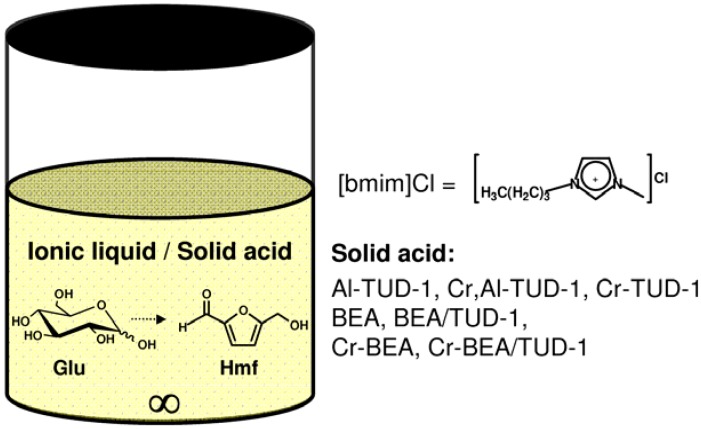
Conversion of d-glucose (Glu) into 5-(hydroxymethyl)-2-furaldehyde (Hmf) using a solid acid/[bmim]Cl catalytic system.

The batchwise dehydration of Glu to Hmf was investigated in the presence of the prepared micro/mesoporous materials as catalysts, using [bmim]Cl as IL solvent, at 120 °C (experiment denoted Catalytic BR, where BR stands for Batch Run) (Scheme 1). This IL was chosen since it solubilises saccharides quite well, possesses a relatively low melting point (ca. 73 °C), is readily available and relatively cheap; furthermore, it has been used successfully as solvent in the homogeneous catalytic reaction of Glu and related di/polysaccharides to Hmf [7,8,9]. The reaction of Glu using [bmim]Cl as solvent, without adding a catalyst, gave 1% Hmf yield at 120 °C/3 h; these poor results are comparable with those reported in the literature for the same reaction using [emim]Cl as solvent, without adding a catalyst, at 100 °C (<5% Hmf yield) [4,44].

For the reaction of Glu using the prepared solid acid/IL systems, conversions in the range 42–96% are reached at 120 °C/3 h (Table 2-Catalytic BR). The formation of Hmf was observed for all catalytic systems (9–58% yield); byproducts included fructose and/or mannose (less than 12% total yield). Levulinic acid was not detected, which may be partly due to the moderately anhydrous conditions of the IL medium (the reaction of Hmf with water can give levulinic acid). The influence of the type of solvent (water, dimethylsulfoxide (DMSO)) on the reaction was investigated for CrAl-TUD-1, under similar reaction conditions to those applied for [bmim]Cl as solvent (120 °C, 3 h). For the three tested solvents, Glu was always completely dissolved in the reaction medium. For water and DMSO, the reaction of Glu was very sluggish: 3% Hmf yield at 28% conversion for DMSO; 2% conversion at 3 h and no Hmf was detected for water as the solvent. These results are much poorer than those observed for the CrAl-TUD-1/IL system (54% Hmf yield), suggesting that [bmim]Cl is a favourable solvent for the target reaction studied. For Al-TUD-1, the use of IL as solvent under moderate conditions did not improve the Hmf yields in comparison to that reported in the literature for a similar material (Al-TUD-1, Si/Al = 21) tested as catalyst in the same reaction using a biphasic water-toluene solvent system, at 170 °C (<20% Hmf yield at 170 °C) [32].

**Table 2:** Catalytic results for the reaction of glucose in the presence of the prepared materials, using [bmim]Cl as solvent, at 120 °C (Catalytic BR), and related catalytic tests for investigating catalyst stability (experiments (i), (ii) and (iii)) ^a^.

Sample	Catalytic BR		Experiment (i)		Experiment (ii)		Experiment (iii)	
	Conv. ^b^ (%)	Yield ^c^ (%)	Conv. ^b^ (%)	Yield ^c^ (%)	Conv. ^b^ (%)	Yield ^c^ (%)	Conv. ^b^ (%)	Yield ^c^ (%)
Al-TUD-1	65	9	23	1	64	9	61	9
CrAl-TUD-1	82	54	37	9	70	12	79	59
Cr-TUD-1	42	39	18	5	42	24	46	42
BEA	85	13	11	4	80	17	75	15
Cr-BEA	96	58	39	10	84	28	96	60
BEA/TUD-1	75	11	34	4	69	15	70	7
Cr-BEA/TUD-1	65	36	19	2	62	13	66	58

^a^ Experiments (i) and (ii) are relative to the recW and recC solids, respectively; experiment (iii) is relative to the recIL liquid (details given in section 2.2.2); ^b^ Glu conversion at 3 h reaction; ^c^ Hmf yield at 3 h reaction.

A comparative study for the different solid acid/IL systems shows that the catalytic systems without chromium lead to relatively high Glu conversions, but low Hmf yields (9–13% yield) compared to those observed for the related chromium-containing systems (36–58% Hmf yield), Table 2. In the case of Al-TUD-1 and CrAl-TUD-1, which possess similar Si/Al ratios and texture properties, CrAl-TUD-1 leads to a much higher Hmf yield. In parallel with these results, the Cr-BEA/IL and Cr-BEA/TUD-1/IL systems lead to higher Hmf yields than the related BEA/IL and BEA/TUD-1/IL systems, respectively. Similar results were reported in the literature for the reaction of Glu in the presence of a hydroxyapatite-supported chromium chloride, using [bmim]Cl as solvent (denoted Cr-HAP/IL system), in that higher Hmf yields were reached than the related system without chromium (denoted HAP/IL), under similar reaction conditions: 40% and 8% yield for Cr-HAP/IL and HAP/IL, respectively, at 78–81% conversion (microwave heating, 400 W, 2–3 min) [23]. The Hmf selectivity for the Cr-TUD-1/IL system was very high (>90% at 42% conversion, Table 2). Hence, while Brönsted acid sites seem to account for enhanced reaction rate of Glu, they are poorly selective in the conversion of Glu to Hmf (favouring side-reactions). Poor catalytic results have been reported in the literature for the reaction of Glu in the presence of a mixture of Brönsted acid and transition metal-containing catalysts, namely a (phosphotungstic acid and chromium)-containing metal-organic framework MIL-101, using [bmim]Cl as solvent (2% Hmf yield, 21% conversion, at 100 °C/3 h); it was postulated that the reaction was Brönsted acid-catalysed [25]. Previously investigated IL-based homogeneous catalytic systems possessing Brönsted acidity, such as H_2_SO_4_/[emim]Cl [4], Brönsted acid-functionalised ILs [45], and Brönsted acid ILs coupled with (Lewis acid) CrCl_3_ [46], were poorly effective in the conversion of Glu to Hmf. According to the literature, the mechanism of the reaction of Glu to Hmf using chromium chloride salt/[emim]Cl, at 100 °C, involves coordination chemistry between Lewis acid chromium species and Glu, accounting for a hydride transfer reaction leading to the isomerisation of Glu into fructose, which is an important primary step in the conversion of Glu to Hmf [4,47].

#### 2.2.2. Catalyst Stability

In order to investigate the stability of the prepared materials in the IL medium, and to assess the homo/heterogeneous nature of the catalytic reaction, each of the materials was firstly put into contact with fresh IL under similar reaction conditions to those used for the Catalytic BR experiments, but without adding Glu; after stirring for 3 h at 120 °C, the solid was separated from the IL by centrifugation, washed with milli-Q water and dried at 55 °C overnight, and separate experiments were carried out with this solid and the recovered IL:

(i) The washed and dried solid, referred to as recW(solid acid), was tested in the reaction of Glu, using fresh IL as solvent, at 120 °C for 3 h;
(ii) The recW solid was calcined (450 °C, 3 h, heating rate of 1 °C·min^−1^) to give recC(solid acid), which was tested in the reaction of Glu, using fresh IL as solvent, at 120 °C for 3 h;
(iii) The recovered IL, referred to as recIL(“name of solid”), was used as solvent in a 3 h-batch run of the reaction of Glu, at 120 °C, without adding a solid catalyst.

**Figure 5:**
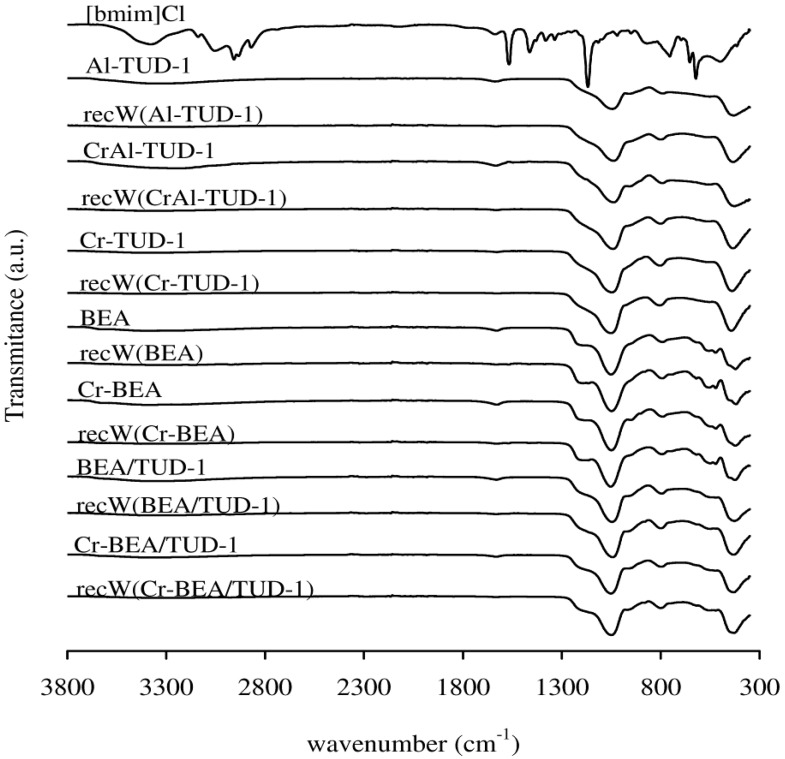
FT-IR ATR spectra of prepared materials and the respective recW solids.

**Figure 6:**
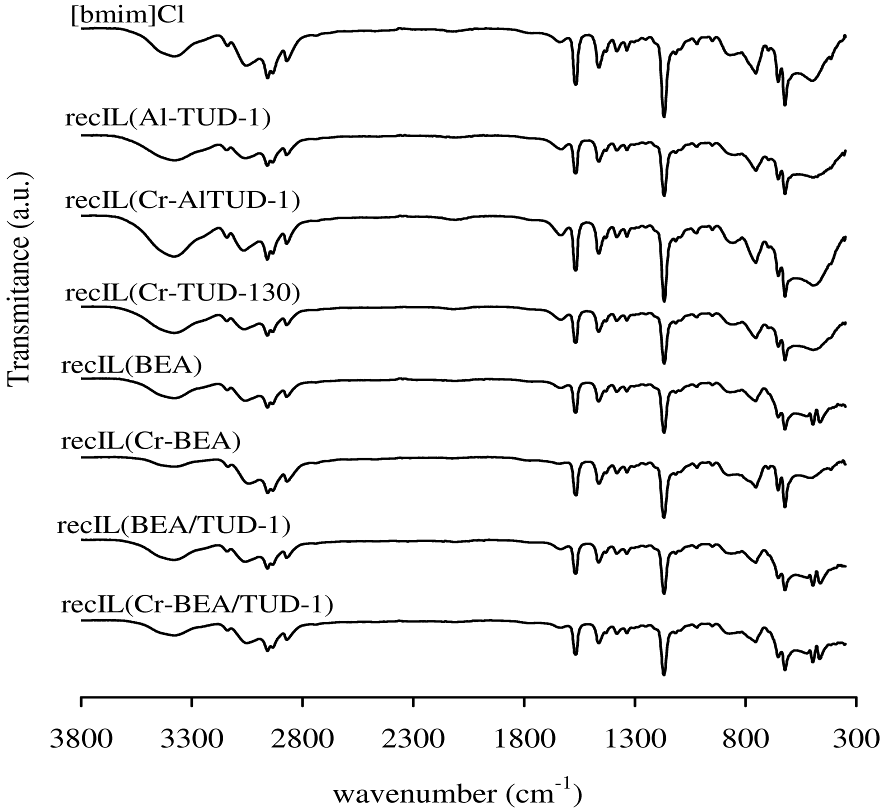
FT-IR ATR spectra of fresh [bmim]Cl and the recIL phases for the different solid acid/IL systems [denoted recIL(name of solid acid)].

No drastic changes in the texture parameters (S_BET_, Vp and PSD) are observed for the fresh and recC solids (Table 1). The powder XRD patterns of the recovered BEA-related solids were similar to those of the respective fresh materials (Figure 1). Hence, the prepared materials seem to possess fairly good microstructural stability in the IL medium. The FT-IR ATR spectra of the recW solids were fairly similar to those observed for the fresh solid acids, and did not show bands characteristic of the IL (Figure 5). The FT-IR ATR spectra of all the recIL phases were quite similar to that of fresh [bmim]Cl, suggesting that this IL is relatively stable under the applied reaction conditions (Figure 6). The DR UV-vis spectrum of recC(Cr-BEA) is comparable to that for Cr-BEA (Figure 4), suggesting that the chemical nature of the surface chromium species is similar. Major differences were observed for recC(Cr-BEA/TUD-1), suggesting that modifications of the surface chromium species occurred (Figure 4). For recC(CrAl-TUD-1) and recC(Cr-TUD-1) the spectra show three bands in similar ranges of wavelengths to those observed for the respective fresh solids, although some changes in the relative intensities are observed, which may be partly due to differences in the relative amounts of the surface chromium species (Figure 4).

The catalytic results for experiment (i) were poorer than those observed for the corresponding Catalytic BR experiment: the reaction of Glu was slower and Hmf yields were lower, especially for the chromium-containing systems (Table 2). The observed catalyst deactivation may be partly due to (a) poisoning of the active sites and/or (b) metal leaching. In order to get insights into hypothesis (a), the catalytic results for experiments (i) and (ii) are compared (Table 2). For the recC solids related to the materials without chromium, the catalytic results are similar to those observed for the Catalytic BR experiments, suggesting that the applied thermal treatment fully activated the recW solids. For the recC solids related to the chromium-containing materials the thermal treatment led to enhanced conversions of Glu, similar to that observed for the remaining solid acids; however, the Hmf yields continued lower than those observed for the corresponding Catalytic BR experiments (slight improvements were solely observed in the cases of recC(Cr-BEA) and recC(Cr-TUD-1)). These results suggest that (selective) chromium species are partially leached from the solids into the IL medium (hypothesis (b)). In fact, ICP-OES analyses for chromium in the recC solids gave residual amounts of chromium in all cases excluding recC(Cr-TUD-1) (Si/Cr ratio of 261 compared to 150 for the respective fresh material). In contrast, the Si/Al ratios are comparable for the fresh and recovered solids, suggesting that the prepared materials are fairly stable towards aluminium leaching in the IL medium: the Si/Al ratios for recC(CrAl-TUD-1), recC(Cr-BEA) and recC(Cr-BEA/TUD-1) were 30, 13 and 45, respectively, compared to 27, 13 and 41 for the respective fresh materials (Table 1). According to the above discussion (Section 2.2.1) related to the roles of Brönsted and chromium Lewis acid species in the reaction of Glu, the (thermally) activated sites may be essentially of Brönsted acid type (active, albeit poorly selective).

**Figure 7:**
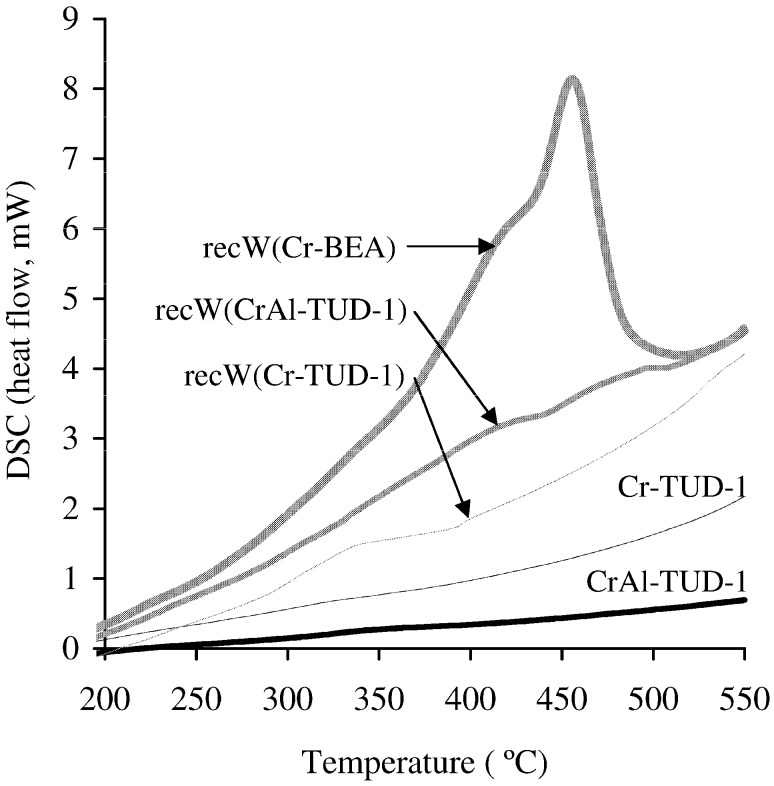
DSC curves for the recW solids related to the chromium containing solid acids (for comparative purposes, exemplified for fresh Cr-TUD-1 and CrAl-TUD-1).

Thermal analyses (TGA and DSC, under similar conditions) were carried out for recW(Cr-BEA), recW(Cr-TUD-1) and recW(CrAl-TUD-1). The DSC curves for the fresh and recovered solids exhibited endothermic bands at temperatures lower than 175 °C assignable to desorption of physisorbed water (Figure 7). Exothermic bands were observed at temperature higher than 300 °C only for the recW solids, which may be due to the decomposition of organic matter; the latter may contribute to the observed catalyst deactivation for recW/IL systems (hypothesis (a) discussed above). Based on the TGA data, the contents of organic matter are 1.2, 2.9 and 8.1 wt.% for recW(Cr-TUD-1), recW(CrAl-TUD-1) and recW(Cr-BEA), respectively. The type of organic matter in the recW solids is most likely related to the cation [bmim]^+^ since the analysed recW solids were recovered from the solid acid/IL mixtures without Glu. It is possible that organic cations in the recW solids are converted to Brönsted acid sites upon the thermal activation treatment, accounting for the improved conversions of Glu observed for the recC solids (without enhancing Hmf yields) (Table 2). 

In order to get insights into the homo/heterogeneous nature of the catalytic reactions using the solid acid/IL systems, a comparative study of Catalytic BR and experiment (iii) was performed. In general, the catalytic results for these two experiments are comparable, suggesting that the catalytic reactions are essentially homogeneous in nature. The active soluble species may be Brönsted acids and/or chromium Lewis acids.

**Figure 8:**
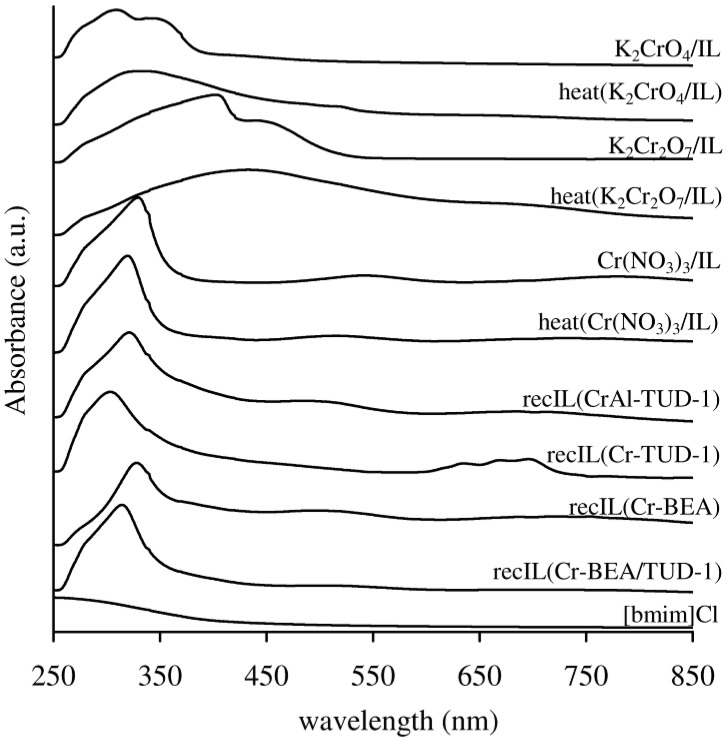
UV-vis spectra of the recIL phases related to the chromium-containing solids, and of chromium salts dissolved in the IL ([bmim]Cl) before and after heating at 120 °C for 3 h.

In order to get insight into the type of soluble active species, the reaction of Glu was carried out in the presence of potassium chromate (K_2_CrO_4_) or dichromate (K_2_Cr_2_O_7_: Used in amounts equivalent to that of chromium added with the CrAl-TUD-1 solid acid), dissolved in the IL (7.2 mM chromium salt). For the two salt/IL systems the reaction of Glu was very sluggish: <1% Hmf yield at 30–36% conversion, 120 °C, 3 h reaction. These results suggest that the active soluble species are not oxochromium(VI) species. Possibly, fully dissociated chromium ions are leached into the IL to give chromium chlorocomplexes. Using Cr(NO_3_)_3_·9H_2_O instead of the chromate salts gives an outstanding 79% Hmf yield at 97% conversion, under similar catalytic reaction conditions. Figure 8 shows the UV-vis spectra of the recIL phases related to the solid acids, and for comparison those of freshly prepared salt/IL solutions and the respective solutions obtained after treatment under similar reaction conditions to those used for the Catalytic BR experiments, but without adding Glu (denoted heat(salt/IL)). The spectra of Cr(NO_3_)_3_/IL and heat(Cr(NO_3_)_3_/IL) are similar, suggesting that the dissolved chromium species are fairly stable under the applied reaction conditions. The same does not apply for K_2_CrO_4_ and K_2_Cr_2_O_7_, suggesting that the dissolved species are not stable under the catalytic reaction conditions. The spectral features of recIL(CrAl-TUD-1), recIL(Cr-BEA) and recIL(Cr-BEA/TUD-1) resemble somewhat more closely those for the Cr(NO_3_)_3_ related systems than those for the K_2_CrO_4_ and K_2_Cr_2_O_7_ related systems; in the case of recIL(Cr-TUD-1), relatively intense bands appear in the region 600–720 nm. These results suggest that the recIL phases contain active soluble Cr^III^ species. Based on the characterisation results in section 2.1 it is not possible to confirm the presence of Cr^III^ in the solid acids prepared, although this possibility cannot be fully ruled out. The active soluble species may result from direct leaching of Cr^III^ and/or from the transformation of leached metal species. 

## 3. Experimental

### 3.1. General

All reagents, solvents and standards were obtained from commercial sources and used as received: aluminum(III) isopropoxide (98%), tetraethylammonium hydroxide (TEAOH, 35 wt.% in water), tetraethylorthosilicate (TEOS, 98%), dimethylsulfoxide (DMSO, ≥99.9%), 1-butyl-3-methyl-imidazolium chloride ([bmim]Cl, ≥98%) and d-glucose from Aldrich, triethanolamine (TEA, 97%) and chromium nitrate (Cr(NO_3_)_3_·9H_2_O, 99%) from ACRÓS, isopropanol (99%) and ethanol (99.8%) from Riedel-de Haën.

ICP-OES measurements for Si, Al and Cr (error ca. 5–10%) were carried out using a Horiba Jobin Yvon Activa M spectrometer, at the Central Laboratory for Analysis, University of Aveiro. Powder X-ray diffraction (XRD) data were collected at room temperature on a Philips X’Pert MPD diffractometer, equipped with an X’Celerator detector, a graphite monochromator (Cu-Kα X-radiation, *λ* = 1.54060 Å) and a flat-plate sample holder, in a Bragg-Brentano para-focusing optics configuration (40 kV, 50 mA). Samples were step-scanned in 0.04° 2*θ* steps with a counting time of 6 s per step. High-resolution transmission electron microscopy (HRTEM) was carried out on Hitachi H9000 and JEOL 2200FS instruments; the sample was prepared by spotting holey amorphous carbon film-coated 400-mesh copper grids (Agar Scientific, Stansted, UK) with a suspension of the solid sample in ethanol. The following textural parameters were estimated from N_2_ adsorption isotherms measured at −196 °C using a Micromeritics Instrument Corp Gemini model 2380 (sample pre-treatment: 250 °C, vacuum): BET specific surface area (S_BET_, calculated for relative pressures (p/p_0_) in the range 0.02–0.10), pore volume (V_p_, using the Gurvitch equation for p/p_0_≅ 0.98), and BJH pore size distribution curve (PSD) calculated from the adsorption branch of the isotherm. Thermogravimetric analysis (TGA) and differential scanning calorimetry (DSC) were carried out under air, with a heating rate of 10 °C·min^−1^, using Shimadzu TGA-50 and DSC-50 systems, respectively. The diffuse reflectance UV-vis (DR UV-vis) spectra were recorded using a Jasco V-560 spectrophotometer and BaSO_4_ as reference. Attenuated total reflectance (ATR) FT-IR spectra were measured on a Mattson 7000 FT-IR spectrometer equipped with a Specac Golden Gate Mk II ATR accessory having a diamond top-plate and KRS-5 focusing lenses.

### 3.2. Preparation of Al-TUD-1, CrAl-TUD-1 and Cr-TUD-1

The aluminium and/or chromium-containing mesoporous silicas of the type TUD-1 were prepared by hydrothermal synthesis. In particular, Al-TUD-1 was prepared as described previously [20]. Specifically, TEOS (17.3 g, 83.0 mmol) was added to aluminium (III) isopropoxide (0.68 g, 3.33 mmol) dissolved in a mixture of isopropanol (6.5 mL) and ethanol (6.5 mL); tomic ratio Si/Al ≅ 25. After stirring for a few minutes, a mixture of TEA (12.51 g, 83.9 mmol) and water (9.4 g) was added, followed by addition of TEAOH (35 wt.% in water, 11.12 mL, 27.0 mmol) under vigorous stirring. The clear gel obtained was stirred at room temperature for 24 h and dried at 98 °C for 24 h, followed by hydrothermal treatment in a Teflon-lined stainless steel autoclave at 180 °C for 8 h. Finally, the solid was calcined at 600 °C in static air for 10 h (heating rate of 1 °C·min^−1^). 

CrAl-TUD-1 was prepared using the following procedure: TEOS (17.3 g, 83.0 mmol) was added to aluminium(III) isopropoxide (0.509 g, 2.5 mmol) and chromium nitrate (0.333 g, 0.8 mmol) dissolved in a mixture of isopropanol (6.5 mL) and ethanol (6.5 mL); atomic ratios Si/(Al*+*Cr) ≅ 25, Si/Cr ≅ 100, Si/Al ≅ 33. After stirring for 30 min, a mixture of TEA (12.5 g, 83 mmol) and water (5.09 g) was added, followed by addition of TEAOH (35 wt.% in water, 17.07 mL, 41.5 mmol) under vigorous stirring. The clear gel obtained was stirred at room temperature for 24 h and dried at 98 °C for 24 h, followed by hydrothermal treatment in a Teflon-lined stainless steel autoclave at 180 °C for 8 h. Finally, the solid was calcined at 600 °C in static air for 10 h (heating rate of 1 °C·min^−1^) to give a yellow powder.

To prepare Cr-TUD-1, a solution of Cr(NO_3_)_3_·9H_2_O (0.38 g, 0.9 mmol) in water (2 mL) was added dropwise to TEOS (19.91 g, 95.6 mmol); atomic ratio Si/Cr ≅ 100. While stirring, a solution of TEA (14.4 g, 96.5 mmol) in water (3.6 mL) was added dropwise, followed by the dropwise addition of TEAOH (35 wt.% in water, 19.7 mL, 47.8 mmol). After stirring for 2 h, a clear and pale green solution was obtained. The mixture was aged at room temperature for 24 h, dried at 100 °C for 24 h, and subsequently subject to hydrothermal treatment in a stainless steel Teflon-lined autoclave at 180 °C for 8 h. Finally the solid was calcined at 600 °C for 10 h in air (heating rate of 1 °C·min^−1^), to give a yellow powder.

The prepared materials were manually ground using an agate pestle and mortar and subsequently sieved to give a powder with particle sizes of less than 106 μm width.

### 3.3. Preparation of BEA, BEA//TUD-1, Cr-BEA and Cr-BEA/TUD-1

Commercial zeolite ammonia Beta powder (NH_4_-BEA, Zeolyst, CP814) was calcined at 550 °C (1°·min^−1^) in static air for 10 h, to give BEA. To prepare Cr-BEA, an aqueous suspension of NH_4_-BEA (2 g) in 200 mL of 0.01 M Cr(NO_3_)_3_·9H_2_O was stirred at 25 °C for 2 h; the solid was then filtered and the ion-exchange procedure repeated twice. Finally, the exchanged solid was filtered, thoroughly washed with deionised water at 60 °C, and calcined at 500 °C for 6 h in air (heating rate of 1 °C·min^−1^). The final solid was a yellow powder.

The composite BEA/TUD-1 was prepared as described previously using BEA zeolite [37]. A similar procedure was used to prepare the composite Cr-BEA/TUD-1. Firstly, BEA was ion-exchanged with chromium using the same procedure as that described above for Cr-BEA, excluding the final calcination step, giving a green powder. Subsequently, TEOS (5.34 g, 0.025 mmol) was added dropwise to a stirred suspension of this powder (1.00 g) in a mixture of TEA (3.85 g, 25 mmol) and water (2.99 g). Then, TEAOH (35 wt.% in water, 3.45 g, 7.5 mmol) was added to the suspension and stirring was continued for 2 h. The gel was aged at room temperature for 24 h, followed by drying at 100 °C for 24 h. The solid material was transferred to a Teflon-lined autoclave and heated at 180 °C for 8 h under static conditions. Finally, the solid was calcined at 600 °C for 10 h in air (heating rate of 1 °C·min^−1^). The final solid was a yellow powder.

The prepared materials were manually ground using an agate pestle and mortar and subsequently sieved to give a powder with particle sizes of less than 106 μm width.

### 3.4. Catalytic Experiments

Batch catalytic experiments were performed under nitrogen in a tubular glass micro-reactor equipped with a valve for gas purging. In a typical procedure, glucose (Glu, 30 mg), powdered catalyst (30 mg), and [bmim]Cl (0.6 mL) were poured into the reactor. The reaction mixtures were stirred magnetically at 1,000 rpm and heated with a thermostatically controlled oil bath (120 °C). Zero time was taken to be the instant the micro-reactor was immersed in the oil bath. The reaction products were analysed using a Knauer K-1001 HPLC pump, coupled to a Knauer 2300 differential refractive index detector (for sugars) and a Knauer 2600 UV detector (280 nm, for Hmf). A PL Hi-Plex Ca 300 mm × 7.7 mm (i.d.) ion exchange column (Polymer Laboratories Ltd., UK) was used: the mobile phase was milli-Q water; flow rate 0.5 mL·min^−1^; column temperature 80 °C. Authentic samples of reagents and products were used as standards for measuring calibration curves. Conversion (%) was calculated as 100 × (moles of Glu consumed)/(initial moles of Glu), and the Hmf yield (mol%) was calculated as 100 × (moles Hmf formed)/(initial moles of Glu). In order to check the data, the experiments were carried out in duplicate and the mean values were calculated.

## 4. Conclusions

The reaction of d-glucose (Glu) was investigated using aluminium and/or chromium containing micro/mesoporous solid acids coupled with [bmim]Cl as solvent, at 120 °C: The prepared materials were Al-TUD-1, CrAl-TUD-1, Cr-TUD-1, BEA, Cr-BEA and the related micro/mesoporous composite materials BEA/TUD-1 and Cr-BEA/TUD-1. The prepared materials seem microstructurally stable in the IL medium, and the Si/Al atomic ratios were similar for the fresh and recovered solids. The solid acids without chromium can be regenerated by thermal treatment, giving similar catalytic results as the respective fresh solids; nevertheless, for these materials the Hmf yields were rather low (<17%). In contrast, the (chromium-containing solid acid)/IL systems led to relatively high Hmf yields (36–58%), although these materials could not be fully regenerated due to chromium leaching which led to an “irreversible” drop in Hmf yield (12–28%). The catalytic reactions are essentially homogeneous in nature. It was postulated that Brönsted acid species seem relatively active in the reaction of Glu, albeit poorly selective in the conversion of Glu to Hmf; in contrast, chromium species play an effective role in the conversion of Glu to Hmf. The IL is a favourable solvent for this target reaction (in terms of Hmf yields reached) in comparison to water or dimethylsulfoxide. The development of truly heterogeneous catalytic systems based on ILs for the selective reaction of Glu and related di/polyssaccharides to Hmf remains a challenge. 
